# Effect of Exercise on Secondary Sarcopenia: A Comprehensive Literature Review

**DOI:** 10.3390/biology11010051

**Published:** 2021-12-30

**Authors:** Rashmi Supriya, Kumar Purnendu Singh, Yang Gao, Yaodong Gu, Julien S. Baker

**Affiliations:** 1 Faculty of Sports Science, Ningbo University, Ningbo 315211, China; gaoyang@hkbu.edu.hk (Y.G.); guyaodong@hotmail.com (Y.G.); jsbaker@hkbu.edu.hk (J.S.B.); 2 Centre for Health and Exercise Science Research, Sarcopenia Research Unit, Department of Sport, Physical Education and Health, Hong Kong Baptist University, Kowloon Tong 999077, Hong Kong; 3 FEBT, School of Environment, Resources and Development, Asian Institute of Technology, Klong Luang, Pathum Thani 12120, Thailand; purnendusin@gmail.com

**Keywords:** secondary sarcopenia, exercise, literature review, human studies

## Abstract

**Simple Summary:**

Sarcopenia is an inevitable component of aging. It is officially recognized as a muscle disease with an ICD-10-MC diagnosis code that can be used to bill for care in some countries. Sarcopenia can be classified into primary or age-related sarcopenia and secondary sarcopenia. The condition is referred to as secondary sarcopenia when any other comorbidities are present in conjunction with aging. Secondary sarcopenia is more prevalent than primary sarcopenia and requires special attention. Exercise interventions may help in our understanding and prevention of sarcopenia with a specific morbidity Glomerular filtration rate that exercise improves muscle mass, quality or physical function in elderly subjects with cancer, type 2 diabetes, kidney diseases and lung diseases. In this review, we summarize recent research that has studied the impact of exercise on patients with secondary sarcopenia, specifically those with one comorbid condition. We did not discover any exercise intervention specifically for subjects with secondary sarcopenia (with one comorbidity). Even though there is a strong argument for using exercise to improve muscle mass, quality or physical function in subjects with cancer, type 2 diabetes, kidney diseases, lung diseases and many more, very few studies have reported baseline sarcopenia assessments. Based on the trials summarized in this review, we may propose but not conclude that resistance, aerobic, balance training or even walking can be useful in subjects with secondary sarcopenia with only one comorbidity due to the limited number of trials. This review is significant because it reveals the need for broad-ranging research initiatives involving secondary sarcopenic patients and highlights a large secondary sarcopenia research gap.

**Abstract:**

Background: Sarcopenia has been recognized as an inevitable part of aging. However, its severity and the age at which it begins cannot be predicted by age alone. The condition can be categorized into primary or age-related sarcopenia and secondary sarcopenia. Sarcopenia is diagnosed as primary when there are no other specific causes. However, secondary sarcopenia occurs if other factors, including malignancy or organ failure, are evident in addition to aging. The prevalence of secondary sarcopenia is far greater than that of primary sarcopenia and requires special attention. To date, nutrition and exercise have proven to be the best methods to combat this disease. The impact of exercise on subjects suffering from sarcopenia with a specific morbidity is worthy of examination for understanding and prevention. The purpose of this review, therefore, is to summarize recent research that has investigated the impact of exercise in patients with secondary sarcopenia, specifically with one comorbidity. Methods: Pubmed, Web of Science, Embase and Medline databases were searched comprehensively with no date limit for randomized controlled trials. The literature was specifically searched for clinical trials in which subjects were sarcopenic with only one comorbidity participating in an exercise intervention. The most visible comorbidities identified and used in the search were lung disease, kidney disease, heart disease, type 2 diabetes, cancer, neurological diseases, osteoporosis and arthritis. Results: A total of 1752 studies were identified that matched the keywords. After removing duplicates, there were 1317 articles remaining. We extracted 98 articles for full screening. Finally, we included 21 relevant papers that were used in this review. Conclusion: Despite a strong rationale for using exercise to improve muscle mass, quality or physical function in subjects with cancer, type 2 diabetes, kidney disease, lung disease and many more, baseline sarcopenia evaluation has been reported in very few trials. The limited number of studies does not allow us to conclude that exercise can improve sarcopenia in patients with other comorbidities. This review highlights the necessity for wide-ranging research initiatives involving secondary sarcopenic patients.

## 1. Introduction

Sarcopenia is a progressive, generalized skeletal muscle illness characterized by accelerated loss of muscle mass and function, which is associated with increased decline in function, frailty and mortality [1]. The condition can be categorized into primary or age-related sarcopenia and secondary sarcopenia. Sarcopenia is diagnosed as primary when there are no other specific causes except aging. The disease is characterized by mitochondrial dysfunction, satellite cell abnormalities, neuromuscular regression, insufficient anabolic hormone production or reduced sensitivity, and the anorexia of aging [2]. However, secondary sarcopenia occurs if other factors, including malignancy or organ failure, are evident in addition to the aging process [1,3]. To mitigate secondary sarcopenia, secondary causes must be appropriately treated, and in elderly populations, sarcopenia has different etiologies and management. Thus, the classification of sarcopenia into primary and secondary can be useful for preventing and treating the disease.

The prevalence of secondary sarcopenia is far greater than that of primary sarcopenia and requires special attention. A study by Therakomen et al. investigating an elderly Thai community reported a prevalence of sarcopenia of 10% based on the 2014 and 2019 Asian Working Group for Sarcopenia (AWGS) criteria [4]. This study excluded patients with secondary sarcopenia. However, in a further study conducted on a community-dwelling of a Thai elderly group using the 2014 AWGS criteria to diagnose sarcopenia, the study reported that the prevalence was 30.5% [4]. The reasons for the discrepancy may be that the later study included patients with type 2 diabetes (T2D) and that T2D complicated by organ failure was one of the causes of secondary sarcopenia [5]. With regard to outpatient settings, a similar study in Asian populations using the AWGS criteria demonstrated that the prevalence of sarcopenia was higher than in the Therakomen et al. [4] study (24%). This may be because patients with T2D were enrolled in the study, and almost 20% of the participants were diagnosed with chronic kidney disease (CKD) [6]. Therefore, the evidence indicates that secondary sarcopenia is more prevalent than primary sarcopenia and needs comprehensive identification and attention.

No medical intervention has been explored or identified to treat this disease. In a review published in 2018, the effects of diet and exercise interventions on changes in lean mass and/or functional outcomes in individuals with sarcopenia or frailty were evaluated [7]. The study’s findings revealed that protein supplementation improved strength and/or functional outcomes; however, other dietary methods did not achieve the same results. Compared to diet and exercise interventions alone, exercise interventions led to consistent improvements in lower body muscle strength but only had a limited effect on walking speed and grip strength [7]. Body composition did not change significantly with lifestyle interventions, excluding calorie restriction [7]. The number of trials that specifically targeted sarcopenic older adults was limited, and more research is needed to determine the type of interventions that will be most appropriate for this high-risk population.

Exercise programs that focus on improving muscle mass and function may be vital in reducing sarcopenia. In a systematic review published in 2019, de Mello et al. examined the effects of physical exercise programs versus no exercise intervention on sarcopenia features and its determinants in sarcopenic elders [8]. The authors reported on five studies investigating the efficacy of isolated exercise programs on the elderly with sarcopenia versus no physical intervention. In all included trials, resistance exercise training (RET) was the primary intervention compared to inactive controls (mainly health education). Comparing training groups with inactive controls, the training groups improved in muscle strength, muscle quality and muscle function. No differences were observed in terms of muscle mass. Randomized control trials (RCTs) were heterogeneous and few, limiting robust conclusions and meta-analysis examination. RET protocols can improve muscle strength and physical performance in elderly people who previously suffered from sarcopenia [8]. 

Among the five studies included in the de Mello et al. systematic review mentioned above, only one study incorporated comorbid subjects and reported that the impact of exercise on subjects with comorbidity taking medications is greater than that of normal sarcopenic subjects [8]. However, they reported cumulative not individual comorbidity and the effects of exercise. All four studies included in the review indicated that most of the sarcopenic subjects were excluded due to the presence of a comorbidity [8]. This indicates that sarcopenic subjects with comorbidities are large and, to a certain extent, are being overlooked. Few investigations have properly addressed this issue by designing good methodological research studies in which the effects of exercise were considered in subjects who were included based on sarcopenic evaluation at baseline along with any specific comorbidities. Therefore, we aimed to unravel the impact of exercise irrespective of its type on secondary sarcopenia with one disease. We selected kidney disease, cancer, T2D, cardiovascular disease, liver, lung, neurological diseases, osteoporosis and osteosarcopenia in association with sarcopenia for our review.

## 2. Search Strategy

The search was completed on 13 October 2021. Pubmed, Web of Science, Embase and Medline databases were searched comprehensively with no date limit used. The literature were specifically searched for RCTs in which subjects were sarcopenic with only one comorbidity engaged in an exercise intervention. The comorbidities included lung disease, kidney disease, heart disease, T2D, cancer, neurological diseases, osteoporosis and arthritis. Sarcopenia with obesity or sarcopenia with metabolic syndrome were not included in this review because they are usually reported in conjunction with other comorbidities and were not included in our inclusion criteria. Key words used in the selection of literature included sarcopenia and exercise or physical activity or training and were individually searched for comorbidities, including cancer or cachexia; lung disease or pulmonary diseases or asthma or cardiac obstructive pulmonary diseases; heart disease or heart failure (HF) or cardiovascular risk factors; kidney disease or renal disease; T2D or diabetes mellitus; liver disease or hepatic disease; osteosarcopenia or osteoporosis or arthritis; and neurological diseases or dementia or Alzheimer’s disease. Exclusion criteria included: reviews, systematic reviews, meta-analysis, observational studies, books, animal studies, papers not written in English and sarcopenia with more than one comorbidity.

## 3. Results and Discussions

A total of 1752 studies were found that matched with our keywords. After removing duplicates, 1317 articles remained. We extracted 98 articles for full screening. Finally, we identified 21 relevant papers that were included in this review.

### 3.1. Effect of Exercise in Patients with Sarcopenia and Cancer

After screening, we found 5 articles that measured baseline sarcopenia with cancer (Table 1). The effect of exercise on subjects with sarcopenia with prostate cancer, breast cancer, metastatic breast cancer, gastric cancer and rectal cancer has been explored, but only on 14 to 53% of baseline sarcopenic subjects. Only one paper by Yamamoto et al. [9], explored the effects of a preoperative exercise program that included handgrip training, walking and RET on subjects who were gastric cancer patients with a diagnosis of sarcopenia based on EWGS. They reported that handgrip strength significantly increased, four patients became non-sarcopenic, but postoperative complications were observed in 3 patients (13.6%); however, none of these complications were severe. This observation requires further investigation using larger sample sizes. The study reported here used a small sample size and was specific to the Japanese population aged ≥ 65 years of age. Another study reported that RET significantly improved lean mass, sarcopenia prevalence, body fat % and strength [10]. By exercising 3 times per week during chemotherapy, resistance and aerobic exercise significantly changed skeletal mass index (SMI) and RET, compared to usual care and aerobic exercise training (AET) combined significantly reversed sarcopenia and dynapenia. Reversal of both sarcopenia and dynapenia was achieved with RET during adjuvant chemotherapy, associated with clinically meaningful improvements in quality of life (QoL) [11]. Sarcopenic compared with non-sarcopenic participants, unsupervised, personalized, 6-month physical activity intervention with activity tracker showed significant changes in muscle cross-sectional area, skeletal muscle ratio density, lean body mass and malondialdehyde [12]. In a telephone-guided graduated walking program that lasted 13–17 weeks, 65% of patients in the pre-rehabilitation group maintained or increased their muscle mass, while 35% experienced a decrease [13]. While there was less than 50% prevalence of sarcopenia at baseline in any of the trials, it is evident that guided walking for 3 to 4 months improved sarcopenia characteristics in patients with cancer.

Patients undergoing cancer treatment can increase muscle mass and strength and improve their physical function by exercising. Researchers conducted a systematic review to examine the effects of physical exercise (aerobic and resistance or combination of both) on strength and muscle mass in cancer patients of different types and at different stages. A total of 16 RCTs were identified for final data synthesis [14]. According to the studies, aerobic and resistance exercise caused greater improvements in upper and lower body muscle strength than the usual treatments. Only a few studies have examined the effects of exercise on muscle mass. Most studies were conducted on patients with early-stage breast or prostate cancer. An isolated study demonstrated that physical exercise increases muscle strength and mass in advanced cancer patients, but further evidence is needed [14].

Prior research has explored the molecular pathway of action and impact of exercise on cancer patients’ tumor progression, muscle atrophy and survival. Some of the mechanisms proposed to explain the potential effects of exercise on cancer progression include the metabolic modulation of glucose-insulin homeostasis, hormonal regulation (testosterone), immune defense, and oxidative damage reduction [15]. In addition, intense exercise can trigger an inflammatory response. When RT practitioners exercise intensively, levels of nuclear factor of kappa light polypeptide gene enhancer in B-cells inhibitor, alpha decrease, while levels of *p*-nuclear factor kappa-light-chain-enhancer of activated B cells (NF-kB) increase within 2 h of exercise and return to near-basal levels within 4 h. Moreover, circulating levels of monocyte chemoattractant protein 1, interleukin-6(IL-6), and interleukin-8 mRNA are significantly upregulated 2 h after exercise. During the first hours following resistance exercise, intense resistance exercise activates NF-kB signaling in human skeletal muscle [16]. Cancer cachexia remains an obscure field when it comes to the contribution exercise can make to patients’ lives. Despite this, the numerous systemic and local benefits of exercise are already being identified [17]. Exercising produces several muscle cytokines, including IL-6, which reduces pro-inflammatory cytokines and increases insulin sensitivity [18].

Consequently, in order for an exercise training program to be appropriate for patients with cancer, it must meet a number of different criteria, including: prescription aligned according to the cachexia stage of the patient; motivation of the patient to adhere to the program; and adequate and constant control of training variables so as not to exceed the patient’s physical condition [19].

### 3.2. Effect of Exercise in Patients with Osteosarcopenia and Osteoporosis with Sarcopenia

The screening process yielded 11 articles that evaluated the effects of exercise on osteosarcopenic subjects (Table 2). There were no subjects participating in the studies under the age of 60. Furthermore, elastic band resistance training was designed to be used three times per week for 12 weeks [20], a periodized single set protocol was scheduled for HIRT twice a week, 12 months [21], 16 months [22], 18 months [23], RET 12 weeks [24], high intensity dynamic resistance exercise (HIT-DRT) for 16 months [25], HIT-DRT for 23 weeks [26], RET performed with 1 or 3 sets for 12 weeks [27], and 18 months of periodized, high-velocity/intensity/effort progressive RET have been explored [28]. These exercises were reported to be beneficial to osteosarcopenic subjects in 10 out of 11 studies.

According to our findings, only high-intensity resistance exercise training has been studied with osteosarcopenic patients. A positive effect of RET has been found on physical function, SMI but no effect on bone mineral density (BMD), vitamin D, alkaline phosphatase, C-terminal telopeptides of type I collagen, expression of miR-206 and miR-133 after a 3 month period. Following 6 months of follow-up, it was noted that the effects were diminished. Dynamic resistance training might be the most effective sarcopenia treatment [29]. Dedicated exercise has also been shown to have positive effects on BMD [21] and fragility fractures [30] in postmenopausal women. Contrary to this, few exercise studies have examined male cohorts, and only half were using DRT protocols (reviewed by Kemmler and colleagues [31]).

There were a total of 11 studies, 8 of which were the FrOST study conducted in Germany, which examined the effects of high-intensity resistance training (HIT-RT) on 43 subjects aged ≥ 73 years. They reported results of HIT-RT following 23 weeks [26], 12 months [21], 16 months [22] and 18 months [23]. The FrOST study also reported on the detraining effect on muscle quality in community-dwelling older men (*n* = 43, age = 78 ± 4 years). Subjects were randomly allocated to a consistently supervised HIRT twice a week and subjected to 6 months of detraining. Their results indicated that six months of absence from HIRT induced a significant deleterious effect on muscle quality in older osteosarcopenic men. They concluded that intermittent training programs with training breaks of 6 months and longer should be replaced by largely continuous exercise programs, at least when addressing muscle quality parameters [32]. Another detraining study by FrOST was conducted on community-dwelling men (*n* = 43, age ≥ 72 years and older with osteosarcopenia) were participants in an 18-month HIRT group or a non-training group [23]. The results showed that HIRT decreased more markedly during the detraining period than during training, though this difference was not significant for lumbar-spine BMD, total-hip BMD, handgrip strength or gait velocity. HIRT group members’ handgrip strength and gait velocity values decreased significantly during detraining, in addition to the hip BMD score. Overall, skeletal muscle mass index and hip-/leg-extensor strength continued to be affected following 24 months. As a result of the findings, the authors concluded that health care providers should focus on continuous exercise instead of intermittent exercises [23]. One of the reasons why detraining or an abrupt cessation approach was taken by the FrOST group was that many older people are unwilling to exercise frequently [33] due to unfavorable conditions for exercise, such as the emergence of COVID 19 and resultant lockdowns. As a result, time efficiency using a low exercise volume and high intensities might be a key feature of exercise protocols for the prevention or treatment of osteosarcopenia. In summary, the authors concluded that there has been no evidence of negative exercise effects in any of the experiments that used osteosarcopenic subjects.

Bone is a dynamic tissue that is affected by a range of physical and dynamic stimuli, including movement, traction and vibration [34]. These forces are constantly active during locomotion and have a major impact on bone and muscle remodeling, so they should be considered in the management of osteopenia and sarcopenia in older adults [34]. It is not known which type, intensity, duration and frequency of exercises are most appropriate for positively influencing osteosarcopenia, though different types of exercises have been described individually for osteopenia and sarcopenia. Not all exercises are beneficial due to the distinct effects they have on bone and muscle. Exercises, such as cycling and walking, do not show any beneficial effect on bone mineral density in any age group [35], however resistance training (RT) or high-impact physical activity, such as running, does improve bone mineral density [36]. In a similar way, resistant exercise is beneficial for sarcopenia because of its direct effects on muscles [37]. In a recent meta-analysis, the role of exercise on sarcopenia-related outcomes was examined and improvements were reported for muscle mass, strength and physical function [38]. Furthermore, 3 times a week of resistance exercises over a 12- to 24-week period can prevent muscle loss in obese older people on calorie-restricted diets [39]. Researchers have reported that resistant exercises improve self-reported physical function and activities of daily living in older people [40]. Vibrating the whole body transmits a vibrating force to muscles and bones, an intervention that can be beneficial in both osteopenia and sarcopenia [41].

### 3.3. Effect of Exercise in Patients with Sarcopenia and Kidney Disease

After screening, we found 3 articles in which the effects of exercise were evaluated in subjects with sarcopenia and kidney disease (Table 3). The ages of the participants ranged from 30 to 80 years. The studies included hemodialysis patients, as well as non-dialysis CKD patients. Intradialytic RET 3 times per week for 3 months [42], 12 months strength or balance training in conjunction with endurance training [43], RET 3 times per week for 6 months [44] were explored. Intradialytic RET 3 times per week for 3 months increased SMI, functional capacity and reduced sarcopenia to 14.3% and 25% in the moderate-load and high-load intradialytic groups, respectively, compared to the control group, which increased by 10% [42]. Twelve months of strength or balance training in conjunction with endurance training reported that in the balance group, leg and whole-body lean mass increased significantly. In the strength group, they remained unchanged. In both strength and balance groups, whole fat mass decreased significantly. Both strength and balance groups showed significant increases in plasma myostatin levels, but there was a significant difference in favoring the strength group. Muscle mass and physical performance were significantly positively correlated with plasma myostatin at baseline, but the associations weakened after 12 months [43]. RET 3 times per week for 6 months improved sarcopenia status, inflammatory profile and anemia biomarkers after the intervention [44].

In 2020, a meta-analysis was published aiming to determine the impacts of fitness training on death rates, kidney function, physical function and adverse events in patients with non-dialysis CKD. A total of 848 patients were evaluated. Exercise did not significantly reduce mortality from all causes or estimate glomerular filtration rate compared with usual care. Contrary to usual care, exercise training significantly increased peak/maximal oxygen consumption. In addition, patients with non-dialysis CKD benefit from regular exercise by improving their stamina and their walking ability. The effect on leg muscle strength was unclear [45]. In another systematic review and meta-analysis of RCTs, it was found that regular, moderate-to-high-intensity RET could result in increased muscle mass and strength, particularly for trained muscles of patients undergoing dialysis. Dialysis patients may benefit from aerobic training and RET [46]. The number of myofibrils increases with RT, especially the fast-twitch fibers, thus increasing the cross-sectional area of the fibers and thereby increasing muscle mass [47]. In addition to increasing the aerobic metabolism of muscular fibers, aerobic training can increase muscle mass [48] in a similar way to RT, but it takes a longer period of time and more frequent sessions than resistance RT [49,50]. The effect size of RT was greater for grip strength than aerobic training, while both could improve upper and lower limb strength. A RT regimen is known to increase insulin-like growth factor-1 levels, which in turn enhances protein synthesis, increases lean body mass, and eventually improves muscular strength. On the other hand, aerobic exercises increase the muscle’s ability to generate adenosine triphosphate within the mitochondria, a substance crucial to muscular contractions and to the development of muscle strength. The reviews above provide evidence for many future studies that are needed to investigate the types of exercises and intensities of exercise that would be best for sarcopenic individuals with CKD and dialysis patients.

### 3.4. Effect of Exercise in Patients with Sarcopenia and Neurological Diseases

Our screening resulted in only one article in which the effect of exercise was evaluated in subjects with sarcopenia and neurodegenerative disease and included mild Alzheimer’s disease (AD) in Korea. Forty women with mild AD and sarcopenia aged 65–80 years performed elastic RET with a Theraband for 40 min, which reduced depression symptoms and enhanced isometric muscle strength [51]. There were two measurements taken: depression and isometric maximal voluntary contraction of the shoulders, hips, elbows, knees and grip strength. Treatment of mild AD patients with sarcopenia reported effective control of depressive symptoms. Following intervention, the exercise group showed greater isometric muscle strength [51]. In their study, RET was found to be an effective treatment option in relieving depressive symptoms in elderly patients with sarcopenia, particularly in those with Alzheimer’s disease. In addition, the findings should contribute to the prevention of sarcopenia symptoms by increasing trained muscle strength (Table 4).

According to these emerging findings, RET, which aids in the maintenance and augmentation of muscular strength, may trigger beneficial neurobiological processes and be crucial to maintaining good health during the aging process, including the preservation of brain function. While there have been a number of studies examining the effects of endurance exercises and/or endurance training on cognitive performance and brain structure, there has been much less research on RET [52]. The link between leg power [53] and whole-body muscle strength [54] is further reinforced by the finding that higher leg power and whole-body muscle strength are associated with higher cognitive scores on standardized tests. Further, greater handgrip strength is associated with higher scores in general cognitive abilities [55] and higher scores in standardized cognitive test batteries [56,57,58]. Further, after 6 months of progressive RET, increases in dynamic muscular strength (measured by one repetition maximum in different RETs) are related to improvements in cognitive function (according to the AD Assessment Scale–cognitive subscale) [59]. Following 3 months of progressive RT, a study concluded that changes in isokinetic knee extension and knee flexion torques mediated improvements in executive functions [60]. An interesting meta-analysis reported that neither muscle size nor strength were associated with cognition [61], but that both muscle function (e.g., muscular strength) and muscle structure (e.g., muscle size) were associated with cognitive ability [61].

In 2019, a systematic review provided an overview of the effects of RET on cognitive functioning through functional and/or structural brain changes. They reported that RET increased frontal lobe activity and improved executive function. RET also contributed to lower white matter atrophy and smaller lesion volumes [62]. However, there have been few studies investigating the effects of exercise on subjects with sarcopenia or neurological diseases. Consequently, future investigations are needed to identify the underlying neurobiological mechanisms and investigate whether the results can be replicated and extended to other groups, such as older adults with dementia, sarcopenia or dynapenia. Exercising regularly has pronounced health benefits. Structural muscle is the best model to understand physiological adaptations to exercise. A key component of exercise-induced adaptations is enhanced mitochondrial function in muscle. In addition, regular exercise benefits the brain, protecting it from degenerative diseases, such as Alzheimer’s disease, the most common type of age-related dementia, and Parkinson’s disease, the most common type of neurodegenerative motor disorder. While there is evidence that exercise induces signaling between the muscles and brain, we do not yet fully understand the mechanism of crosstalk between the two. Mitochondria, however, play an important role in both organs. An overview of a study examined the role of mitochondria in the pathways from muscle to the brain induced by exercise. Among these routes are circulating factors, such as myokines, whose release is often influenced by the mitochondria, as well as direct mitochondrial transfer [63]. The remodeling of neuromuscular junctions (NMJs), as well as age-related neurophysiological changes may also contribute to neuromuscular impairment [64]. Studies by Nagamatsu et al. [65] confirm the positive impact of RT on brain health and noted that trained seniors with mild cognitive impairment (MCI) performed better on an associative memory test after long-term RT (52 weeks). Additionally, higher cortical activity was positively correlated with improved cognitive performance [65]. RT may also improve brain health by modulating functional connectivity in MCI. Individuals with MCI have been observed to have decreased resting-state functional connectivity between the posterior cingulate cortex and other brain regions [66,67], functional connectivity between the parietal and temporal cortex is related to test performance [66], individuals with Alzheimer’s disease or MCI have impaired resting-state connectivity between the hippocampus and other brain regions [68]. RT lasting 26 weeks increases functional connectivity between the anterior cingulate cortex, the posterior cingulate cortex, the left inferior temporal lobe, and the hippocampus [69]. It is speculated that RT is an effective intervention strategy to enhance brain health and cognitive function by improving resting-state functional connectivity, based on the changes in resting-state functional connectivity in neurological diseases (e.g., MCI). Despite the evidence, studies that demonstrate the benefits of exercise for people with sarcopenia and neurological diseases remain sparse.

### 3.5. Effect of Exercise in Patients with Sarcopenia and Lung Disease

Among the articles screened, only one evaluated the effect of exercise in subjects with 55% of baseline sarcopenia (*n* = 112, age 66 ± 8 years) with lung disease COPD [70] (Table 5). A 4-week high-intensity pulmonary rehabilitation program was conducted. Among the outcomes evaluated were blood biomarkers, SMIs and IRs. Sarcopenia was associated with a significant decline in insulin resistance (IR), fat mass index, waist circumference, and low-density lipoprotein (LDL) compared to non-sarcopenic individuals. Both groups experienced a reduction in total cholesterol levels. Rehabilitation performed on sarcopenic and non-sarcopenic patients was examined for its impact on body composition parameters and cardiometabolic risk factors. Four weeks of high-intensity training were successfully completed by 85 patients. In 50% of both sarcopenic and non-sarcopenic patients, physical performance was improved beyond minimal clinically important differences, and QoL was improved in more than 75%. Lean mass did not change significantly in either group, while body fat and waist circumference decreased significantly among the non-sarcopenic group. At the group level, total cholesterol levels and high-density lipoprotein cholesterol (HDL) levels showed significant declines. LDL cholesterol levels were also reduced in non-sarcopenic patients [70].

Many hypotheses have been proposed to explain how exercise affects muscle in COPD patients [71]. Exercise-based pulmonary rehabilitation was found to reduce exercise-induced lactate levels and ventilation at iso-time during a cycling test, especially in COPD patients who were trained at high work rates [72]. Exercise performance improvements following moderate-to-high intensity exercise training were attributed, at least in part, to changes in physiology at the level of the lower-limb muscles. Following a 12-week aerobic training program in patients with COPD, another study showed that exercise training can result in physiological changes, including an increase in oxidative enzyme activity (e.g., citrate synthase and 3-hydroxyacyl-CoA dehydrogenase) and a reduction in exercise-induced lactate production [73]. As a result, exercise training has been scientifically proven to be an essential part of pulmonary rehabilitation for patients with COPD. Another study documented that exercise training in combination with education enhanced exercise performance significantly more than education alone in 128 patients with COPD [74]. The authors, however, did not examine the metabolic changes in the lower-limb muscles. In COPD patients, whole-body endurance exercise training was a standard part of pulmonary rehabilitation. According to another study, most COPD patients were unable to exercise at a high intensity [75].

Patients with severe COPD gained improved exercise tolerance, dyspnea sensations, functional capacity, and quality of life by undergoing exercise training as part of pulmonary rehabilitation. The problem is that some patients may be unable to tolerate the feeling of breathlessness or peripheral muscle discomfort long enough to achieve physiological training effects. Therefore, there is a major challenge involved in selecting a training strategy that is tailored to individual patient limitations, such as cardiovascular, pulmonary and peripheral muscle limitations, to maximize the effect of exercise conditioning. Interval training and resistance exercises are also particularly important since both modalities permit painful loads to be placed on peripheral muscles with tolerable levels of dyspnea. Performing short intervals of muscle strength conditioning, combined with oxygen breathing, may constitute a viable and effective approach for pulmonary rehabilitation for patients with profound muscle weakness and intense breathlessness during the onset of physical exertion [76]. Hence, more research is needed to emphasize the effect and types of exercise on lung diseases in subjects with sarcopenia.

### 3.6. Effect of Exercise in Patients with Sarcopenia and Cardiovascular Disease

There was only one study, in 2021, that determined if RET can be performed with moderate blood flow restriction (KAATSU RT). Study results showed that KAATSU RT could safely increase muscle strength and size in patients undergoing cardiac surgery (*n* = 21, age = 69.6 ± 12.6 years) (Table 6). The researchers found that anterior mid-thigh thickness, SMI, walking speed, and knee extender strength improved after 3 months of KAATSU RT treatments. The physical function of low-functioning vs. high-functioning patients increased more with KAATSU RT after 3 months. When used in addition to standard cardiac rehabilitation, low-intensity KAATSU RT has been shown to significantly improve skeletal muscle strength and size in cardiovascular surgery patients [77].

Exercise below the anaerobic threshold, at a low intensity, is typically part of cardiac rehabilitation [78,79], which can improve exercise capacity but has only minor effects on muscle strength and mass [80]. Patients with frailty and sarcopenia are at a higher risk for muscular atrophy, but low-intensity aerobic activities alone are ineffective for enhancing muscle strength and mass. As a result, cardiac rehabilitation should include aerobic exercises, as well as RT [81,82]. The American College of Sports Medicine estimates that when performed in 3–4 sets of 8–12 repetitions to fatigue, RT at 60–70% of one repetition maximum is most effective at improving maximal voluntary force and inducing muscle hypertrophy [83]. Nevertheless, elderly patients who undergo cardiac surgery are often not able to perform high-intensity RET after surgery. There is a need to develop and investigate a method of RET that is safe and effective for patients post cardiac surgery, which improves muscle size and strength. A specially designed cuff restricts blood flow moderately to the lower or upper extremities during KAATSU training. By using low-intensity short-term RT, it has been well established that this method will increase muscle strength and size, both in athletes and healthy subjects [84,85]. It is still not clear how KAATSU training enhances low-intensity RT. However, it might be associated with an increase in muscle activation [84,86]. A certain degree of muscle activation was observed in cardiovascular patients with or without cardiovascular surgery with KAATSU RT [87]. Studies have shown that KAATSU RT can cause possible adverse side effects, such as dizziness, subcutaneous hemorrhage, cutaneous hemorrhage, drowsiness, numbness, nausea and itchiness [88]. A number of studies have concluded that KAATSU RT has no adverse effects in rehabilitating older adults [85], in those recovering from anterior cruciate ligament surgery [89], and in those with ischemic heart disease [90]. As a result, KAATSU RT has the potential to be a helpful method for patients undergoing cardiac surgery who want to improve their muscle strength and size post-event. There was also an investigation of the effects of aerobic exercise and RT on carotid arterial intima-media thickness and flow velocity in elderly women with sarcopenic obesity [91]. The RCT of progressive RET has also been used to counteract the myopathy associated with chronic HF [92]. In this regard, we found many trials, but those in which exercise was used and demonstrated to have a positive effect on patients with sarcopenia are lacking and are warranted.

Research has also explored the molecular mechanisms underlying muscular effects of exercise on subjects with cardiovascular diseases, including inflammation, catabolic/anabolic balance, energy metabolism and fiber composition [93]. Several authors reported that, depending on the severity of chronic HF, no change in biomarkers of inflammation was detected in serum [94,95] or skeletal muscle [96,97]. Chronic HF patients often exhibit skeletal muscle atrophy. There is a clear connection between exercise training and the modulation of muscle ring-finger protein-1 and muscle atrophy F-box gene expression. In animal studies [98,99] and human studies [100], both E3 ligases are found to be reduced after exercise training. Muscle mass is negatively affected by myostatin, which modulates muscle growth and differentiation [101]. Further evidence that myostatin modulates muscle size is provided by observations that animals lacking the gene [102] and humans with mutations in both copies of the gene [103] have greater muscle mass. There has only been one study examining how exercise training affects myostatin expression in HF patients. Compared to prior exercise training, exercise training resulted in a 36% reduction in myostatin mRNA expression and a 23% decrease in myostatin protein expression [104]. An energetic imbalance results from an augmented energy demand and a diminished energy metabolism in HF [105,106]. Exercise training affects skeletal muscle metabolism by changing mitochondrial function and capillary supply quantitatively and qualitatively [107,108]. Exercise-induced changes in skeletal muscle energy metabolism are induced by the peroxisome proliferator-activated receptor-gamma coactivator −1 α and other signaling molecules, such as mitogen-activated protein kinase, Ca^2+^/calmodulin-dependent protein kinase and 5′ AMP-activated protein kinase [109,110]. Biopsies of skeletal muscle taken from HF patients often show a shift in fiber type composition when compared to healthy controls. There were reversals of fiber type composition changes and a reduction in the capillary-to-fiber ratio in HF after regular exercise training [111].

### 3.7. Effect of Exercise in Patients with Sarcopenia and Liver Disease

We did not locate an RCT that evaluated exercise for patients with sarcopenia and liver disease. We were able to find only a few studies that investigated the effect of exercise on frailty, muscle mass, quality and physical function in patients with liver disease. In all trials to our knowledge, baseline sarcopenia was not explored.

An article reported the impact of physical exercise on physical frailty in patients with chronic liver disease even after liver transplantation and found that exercise improves aerobic capacity (VO2) peak, anaerobic threshold, 6-min walk distance, muscle mass, and quality-of-life for both compensated and decompensated liver disease patients in 11 studies. With a combination of aerobic and RET at moderate-to-high intensity, the improvements were most significant. The studies did not include patients with significant liver failure, as the number was small (*n* = 1–50) and concentrated on supervised, hospital-based exercises. Four RCT studies and 3 observational studies showed that predominantly supervised aerobic exercise improved aerobic capacity, muscle strength and quality-of-life after liver transplantation. In terms of timing, intensity and type of exercises, there was noticeable heterogeneity. In chronic liver disease and after liver transplantation, exercise can improve key components of physical frailty (functional/aerobic capacity, sarcopenia), as well as quality-of-life. In large controlled clinical trials, future research should be devoted to understanding how exercise type, compliance, intensity and duration impact clinical outcomes [112].

A further interesting review discusses how physical activity and nutrition may lead to improvements in sarcopenia in patients with liver cirrhosis. In cirrhosis, sarcopenia arises from three major factors: insufficient nutrition, metabolic problems and malabsorption. Sarcopenia appears to spare muscles in its early stages, but it is associated with mobility limitations, risk of falling and a significantly reduced quality-of-life. Studies have shown that physical activity and balanced nutrition play an important role in preventing this chronic disorder. It is recommended that patients with these conditions engage in exercise and nutritional interventions to improve their quality-of-life [113]. Relatively little recent research has examined the effects of exercise on chronic liver disease. Most of the research in this field focuses on non-alcoholic fatty liver disease (NAFLD), where mounting clinical and experimental evidence suggests that crosstalk between skeletal muscle and adipose tissue is responsible for regulating intrahepatic fat storage. Physical activity combined with calorie restriction is considered necessary in this condition to effectively reduce intrahepatic lipids, but there is less evidence that vigorous activity improves NAFLD. Despite the paucity of evidence, physical activity ought to be considered an important component in the treatment of patients with liver disease in order to improve their clinical outcomes [114]. There is little evidence that exercise decreases the risk of hepatocellular carcinoma; however, some epidemiological studies suggest that patients who exercise regularly and vigorously have a lower risk. Despite acutely increasing portal pressure in compensated cirrhosis, exercise has been shown to be both safe and beneficial in the long term. The VO2 decrease is associated with mortality in decompensated cirrhosis patients, who are almost always sarcopenic. Physical activity improves VO2 in these patients, reducing hepatic encephalopathy risk through increased skeletal muscle mass. Solid organ transplant recipients benefit from exercise by gaining lean mass, increasing their muscular strength, and thereby increasing their aerobic capacity. Few studies have been conducted on liver transplant recipients, in whom exercise would be a worthwhile subject for future research given its high potential for long-term benefits [114]. It is interesting to note that different types of exercise are beneficial to patients with different types of liver diseases for outcomes such as muscle mass quality or physical function.

Nevertheless, we cannot conclude that exercise can benefit patients with sarcopenia and liver disease, since any baseline sarcopenia measurement by any definition, by any research group, was not considered, and hence requires further research.

### 3.8. Effect of Exercise in Patients with Sarcopenia and Type 2 Diabetes

Our search did not uncover any RCTs examining the effects of exercise on patients with sarcopenia and T2D. Our study revealed, however, a prospective study involving 44 diabetic patients over 70 years old. A strength-training program with an elastic band 3 days a week and 30-min walk 5 days a week were recommended for 6 months. A total of 38.6% of patients did not adhere to aerobic exercises, and 47.7% did not complete exercises with elastic bands. The prevalence of frailty decreased from 34.1% to 25% and the proportion of patients with moderate-to-severe functional limitations decreased from 26.2% to 21.4%. Thus, they concluded that using an elastic band and aerobic exercise reduces frailty among elderly people with T2D. Those with coronary ischemic heart disease are less likely to improve their frailty than those who follow an aerobic exercise routine [115]. Aerobic exercises and elastic band exercises might reduce the prevalence of sarcopenia, though further investigation is still needed as baseline characteristics did not define sarcopenia.

We identified recent reviews exploring the effects of exercise on T2D patients. The systematic review and meta-analysis focused on identifying the effectiveness of structured exercise interventions in T2D. During the analysis, 846 participants were analyzed, of whom 440 were in the intervention group and 406 were in the control group. Based on moderate level 2 evidence, structured exercise interventions for insulin resistance in T2D were effective. Only fasting insulin level, hemoglobin A1c(HbA1c), fasting blood sugar level and body mass index (BMI) were reported [116]. A further systematic review, with 978 participants, provides useful information regarding the clinical implementation of combined exercise in the management of patients with T2D and overweight/obesity at the same time. Patients with T2D and concurrent overweight or obesity benefit from combined exercise intervention in terms of improved glycemic control and weight loss, as well as improved insulin sensitivity [117]. In another systematic review, fitness training was linked to lower body mass and HbA1c in patients with T2D. In the analysis, exercises from 12 aerobic training studies and 2 RET studies were included. Post-intervention means were calculated. In their study, exercise training reduced HbA1c, but body mass did not change significantly more when exercise groups were compared to controls [118]. Despite the positive mechanisms already discovered in favor of diabetic subjects after exercise intervention, the three systematic and meta-analyses studies only reported BMI. They did not report on muscle mass, quality or function by comparing it with baseline sarcopenia as an outcome measure, so further research is needed.

## 4. Conclusions

In studies where exercise interventions were delivered to elderly patients with any one of the diseases investigated, domains for the disease, muscle mass or function have been often reported, but the baseline measurements of sarcopenia for any consensus definition were lacking. However, we were able to explore that RT exerts beneficial effects on subjects with sarcopenia with any other comorbidity, including cancer, kidney, lungs, liver, neurological diseases and osteosarcopenia. However, we did not find any RCT of exercise on subjects with sarcopenia and liver diseases or sarcopenia and T2D. Research has already indicated that exercise exerts favorable impacts on muscle mass, quality and function in liver disease and T2D, but the baseline measurements of sarcopenia are lacking in such interventions and hence the results are difficult to translate.

Despite a strong rationale for the use of exercise, there are few studies that used RCTs to elucidate specific effects in subjects with sarcopenia and specific comorbidities. Due to very few clinical trials and insufficient evidence to determine the safety and effectiveness of exercise for patients with sarcopenia and any other comorbidities, we still cannot conclude on the effectiveness of exercise for secondary sarcopenic subjects. The impact of exercise on subjects suffering from sarcopenia with a specific morbidity is worthy of examination for understanding the disease and the design of preventive strategies. The purpose of this review was to summarize research in which the impact of exercise has been explored in patients with secondary sarcopenia, specifically with one comorbidity.

This review has identified potentially a wide range of new research topics that need consideration for secondary sarcopenic subjects.

## 5. Future Direction

Different types of exercise need to be explored for patients with sarcopenia and specific comorbidities. The exercises may need to be based on comorbidity, not sarcopenia.

Future research that will explore the effect of exercise on muscle mass, quality or physical function in any non-communicable disease with muscle loss phenomenon should consider baseline measurement of sarcopenia based on consensus definitions of sarcopenia suitable and specific for identified populations.

The effects of temporary breaks in exercise routines, e.g., due to the intermittent nature of an exercise program, lack of time or simple reluctance, might be a frequent situation that compromises older adults’ lives and must be explored. Like the FrOST studies investigating osteosarcopenic subjects, the detraining effect of exercise in subjects with sarcopenia and other comorbidities needs to be examined further.

## Figures and Tables

**Table 1:** Effect of exercise on subjects with cancer and baseline measurement of sarcopenia.

S.No	Authors, Year	Place, N	Subjects with (Baseline Sarcopenia)	Mean Age ± SD (Years), Sex	Exercise Intervention and Number of Groups	Measured Outcomes	Results	
1	Dawson et al., 2018	Los angeles, 37	Prostate cancer patients on ADT (43.8% of participants were sarcopenic)	67.4 ± 8.8, M	Resistance training for 12 weeks 4 groups: TRAINPRO, TRAIN, PRO, STRETCH	-body composition, MetS, QoL, physical fitness, Muscular strength	**EXE compared to Non EXE**	
							lean mass	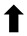
							sarcopenia prevalence	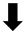
							body fat %	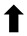
							strength	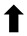
							prostate cancer-specific QoL	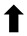
2	Adams et al., 2016	Ottawa, vacuover; 200	Breast Cancer patients (25.5 % were sarcopenic and 54.5 % were dynapenic)	48.8 ± 13.3, F	resistance and aerobic exercise 3 times/week during chemotherapy 3 groups: UC, AET, RET	-QoL, physical function Fatigue, LBM, percent body fat, bone mineral content, muscular strength.	**RET compared UC**	
							SMI	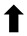
							**RET, compared to UC/AET combined**	
							Prevalence sarcopenia and dynapenia	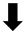
							**RET during adjuvant chemotherapy**	
							QoL	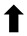
3	Delrieu et al., 2021	Lyon France; 49	metastatic breast cancer patients (53% were sarcopenic and 75% had poor muscle quality.)	55.0 ± 10.4, F	unsupervised, personalized, 6-month physical activity intervention with activity tracker - Single group	-SMI, muscle quality, Oxidative markers including plasma antioxidant enzymes, prooxidant enzymes and oxidative stress damage markers	**Sarcopenic compared to non-sarcopenic**	
							Muscle cross sectional area	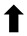
							skeletal muscle radiodensity	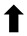
							lean body mass	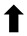
							MDA	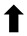
4	Yamamoto et al., 2017	Japan, 22	gastric cancer patients with a diagnosis of sarcopenia (EWGS)	72.7 ± 4.5, M:F = 5:6	preoperative exercise program consisted of handgrip training, walking, and resistance training - Single group	-4-m gait speed testing, handgrip strength testing, SMI	Handgrip strength	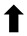
							sarcopenia prevalence	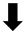
5	Moug et al., 2020	West of Scotland; 44	Rectal cancer patients (14% were sarcopenic)	66.8 ± 9.6, M:F = 7:4	13–17-week telephone-guided graduated walking programme -2 groups: Intervention, control group	-baseline physical assessment, psoas muscle mass measurement	**Intervention compared to control group**	
							muscle mass	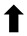

Note: N: Number of subjects, M: Male, F: Female, MetS: Metabolic syndrome, NoEXE: Non-exercise groups, PRO: Protein supplementation group, PSA: Prostate-specific antigen, RCT: Randomized controlled trial, STRETCH: Stretching group, TRAIN: Resistance training group, TRAINPRO: Resistance training and protein supplementation group, QoL: Quality of life, ADT: Androgen deprivation therapy, EXE: Exercise groups, RET: resistance exercise training, AET: aerobic exercise training, UC: usual care, SMI: skeletal muscle index; LBM: Lean body mass; MDA: malondialdehyde.

**Table 2:** Effect of exercise on subjects with osteosarcopenia.

S.No	Authors, Year	Place, N	Subjects	Mean Age ± SD (Years), Sex	Exercise Intervention	Measured Outcomes	Results	
1	Banitalebi et al., 2021	Iran, 63	Osteosarcopenic	64.1 ± 3.6, F	RET via elastic bands 3 times/ week for 12-weeks. -2 group: Intervention, control	-Fracture Risk Assessment Tool score, bone mineral content, bone mineral density, vitamin D, alkaline phosphatase, C-terminal telopeptides of type I collagen, expression of miR-206 and miR-133	No change	
2	Ghasemikaram et al., 2021b	Germany, 43	Osteosarcopenia	78 ± 4, M	The HIRT scheduled a periodized single set protocol 2 times/weekly. After the intervention, the men were subjected to 6 months of detraining -2 groups: HIRT, Control	MQ, maximum isokinetic hip/leg extensor strength per unit of mid-thigh intra-fascia volume	**Detraining effect**	
							MQ	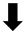
3	Kemmler et al., 2020b	Germany, 43	Sedentary community dwelling subjects with osteopenia/osteoporosis and SMI-based sarcopenia	78.5 ± 4.3, M	HIRT for 12 months -2 groups: HIRT, Control * HIRT provided a progressive, periodized single-set DRT on machines with high intensity, effort, and velocity twice a week, * CG: maintain lifestyle * Both groups: supplemented with whey protein, vitamin D, and calcium	-BMD, SMI	**EG compared to CG**	
							LS-BMD	Maintained
							SMI	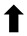
							Total hip BMD	No change
4	Kemmler et al., 2021a	Germany, 43	Community-dwelling men with osteosarcopenia	78.5 ± 4.3, M	HIRT for 18 months -2 groups: HIRT, Control * After intervention of 18 months stopped HIT-RT for 6 months, but continued their habitual physical activity	-SMI, BMD at the lumbar-spine and total-hip -maximum hip/leg-extensor strength, HGS, gait velocity.	**Detraining in the HIT-RT compared to CG**	
							SMI	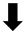
							hip-/leg-extensor strength	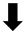
							total-hip BMD	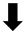
							HGS	No change
							gait velocity	No change
5	Lee et al., 2021	Taiwan, 27	Osteosarcopenic	71.1 ± 4.9, F	RET 12 weeks -2 Groups: experimental (RET) control groups (no exercise)	-Lean mass, physical capacity assessments	**RET compared to CG**	
							physical function	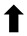
6	Ghasemikaram et al., 2021a	Germany, 43	Sedentary community dwelling subjects with osteopenia/osteoporosis and SMI-based sarcopenia	78.5 ± 4.3, M	HIRT for 16 months -2 groups: HIRT, Control * HIRT provided a progressive, periodized single-set DRT on machines with high intensity, effort, and velocity twice a week, * CG: maintained lifestyle. * Both groups: supplemented with whey protein, vitamin D, and calcium	-muscle, adipose tissue volume, fat fraction of the thigh.	**EG compared to CG**	
							IMAT volume	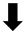
							fat fraction within the deep fascia of the thigh	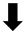
7	Kemmler et al., 2020c	Germany, 43	Osteosarcopenia	78.5 ± 4.3, M	The HIRT scheduled a periodized single set protocol 2 times/ week. After the intervention, the men were subjected to six months of detraining -2 groups: HIRT, Control	-LBM, total and abdominal body fat rate, MetSZ	**HIT-RT compared to CG**	
							LBM	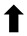
							total and abdominal body fat rate	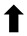
							MetSZ	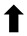
8	Kemmler et al., 2021b	Germany, 43	Sedentary community dwelling subjects with osteopenia/osteoporosis and SMI-based sarcopenia	78.5 ± 4.3, M	HIT-DR for 16 months -2 groups: HIT-DRT, Control * HIT-DRT: Supervised HIT-RT twice/week and whey protein 1.5–1.6 g/kg/body supplementation * CG: maintained lifestyle and whey protein1.2 g/kg/body mass/d * Both groups were supplied with calcium and vitamin D	-BMD, sarcopenia Z-score.	**HIT-DRT compared to CG**	
							sarcopenia Z-score	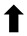
							-total hip	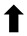
							-BMD at lumbar spine	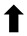
9	Lichtenberg et al., 2019	Germany, 43	Sedentary community dwelling subjects with osteopenia/osteoporosis and SMI-based sarcopenia	78.5 ± 4.2, M	HIT-DRT for 23 weeks -2 groups: HIT-DRT, Control * HIT-DRT: Supervised HIT-RT twice/week and whey protein 1.5–1.6 g/kg/body supplementation * CG: maintained lifestyle and whey protein1.2 g/kg/body mass/d * Both groups were supplied with calcium and vitamin D	-Sarcopenia Z-score, physiological parameters, SMI, HGS, gait velocity.	**HIT-DRT compared to** **CG**	
							sarcopenia Z-score	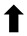
							SMI	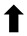
							HGS	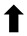
10	Cunha et al., 2018	Brazil, 62	osteosarcopenic	68.0 ± 4.3, F	RET with 1 or 3 sets for 12 weeks -3 groups: G1S(1 set), G3S(3 sets), Control (no)	-Body composition, BMD, SMI	**G3S compared to G1S**	
							body fat	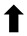
							**G1S and G3S compared to CG**	
							SMI	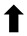
11	Kemmler et al., 2020a	Germany, 43	Osteosarcopenic	78.5 ± 4.3, M	18 months of periodized HIT-RT * single-blind, two-group parallel design * exercise group: supervised periodized HIT-RT on machines 2 times/week * control group: maintained their physical activity/exercise habits.* Both groups were supplied with protein, cholecalciferol, and calcium	-LBM, total and abdominal body fat, maximum hip/leg extensor strength	**HIT-RT vs. CG**	
							LBM	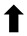
							total body fat mass	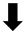
							abdominal body fat percentage maximum hip/leg extensor strength	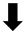 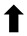

Note—N: number of subjects, HIT-RT: high-intensity resistance exercise training, MQ: Muscle quality, BMD: bone mineral density, HGS: Handgrip strength, intramuscular adipose tissue (IMAT), Metabolic Syndrome Z-Score (MetSZ), M: Male, F: Female, * is the explanation of the interventions.

**Table 3:** Effect of exercise on subjects with sarcopenia and kidney disease.

S.No	Authors, Year	Place, N	Subjects	Mean Age ± SD (Years), Sex	Exercise Intervention, Number of Groups	Measured Outcomes	Results	
1	Lopes et al., 2019	Brazil, 80	Individuals on hemodialysis (prevalence of pre-sarcopenia and sarcopenia was 5% and 30% in the CG; 50% and 25% in the MLG; and 28.6% and 21.4 % in the HLG, respectively)	54.2 ± 12.0, Both M:F = 1:1	The 12 weeks of intradialytic RT 3 times per week -3 groups: HLG: 8–10 repetitions, MLG: 16–18 repetitions, CG: stretching exercise	Body composition, sarcopenia prevalence, functional capacity, inflammatory markers, QoL	**HLG compared to CG**	
							LLM	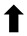
							pain and physical function domains	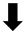
							**Both RT compared to CG**	
							SMI, functional capacity	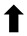
							Prevalence	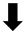
2	Zhou et al., 2021	Lund, 112	non-dialysis-dependent patients with CKD(10% sarcopenia)	67.0 ± 13.0, Both M:F = 2:1	strength or balance in combination with endurance training for 12 months -2 group: Balance training, strength training	Body composition, Plasma myostatin	**Balance compared to strength group**	
							leg and WBLM	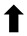
							Prevalence of sarcopenia	No Change in both groups
							Plasma myostatin	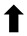
3	Gadelha et al., 2021	Brazil, 107	CKD with sarcopenia	65.4 ± 3.7 Both (ratio not given)	Treatment groups underwent a 24-week intervention with RT before each dialysis session, three times per week. -4 groups: sarcopenic RT, non-sarcopenic RT, sarcopenic control, non-sarcopenic control	Blood sample analysis for ferritin, hepcidin, iron availability, and inflammatory profile (TNFα, IL-6, and IL-10) was conducted. All-cause mortality was recorded over 5 years	**Sarcopenic RT group compared to other groups**	
							sarcopenia status	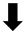
							inflammatory profile and anemia biomarkers	improved

Note—RT: resistance training, QoL: quality of life, HLG: high-load intradialytic group, MLG: moderate-load intradialytic group, CG: control group, LLM: Lean Leg Mass, SMI: skeletal muscle index, CKD: Chronic Kidney Disease, whole-body lean mass: WBLM, M: Male, F: Female.

**Table 4:** Effect of exercise on subjects with sarcopenia and neurological diseases.

S.No	Authors, Year	Place	Subjects	Mean Age ± SD (Years)	Exercise Intervention	Measured Outcomes	Results	
1	Chang et al., 2020	Korea, 40	mild AD and sarcopenia	79.3 ± 5.1, F	40-min elastic resistance exercise using Theraband -2 groups: exercise (3 training sessions in nonconsecutive days /week for 12 weeks. Each session 10-min general warm-up, 40-min elastic resistance exercise using Theraband and cool down), control group did not perform any exercise. routine	-Depression, isometric maximal voluntary contraction shoulder abduction, hip and elbow flexion, knee extension, grip strength, gait speed	**Exercise compared to control group**	
							depressive symptoms	controlled
							isometric muscle strength.	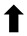

Note—AD: Alzheimer’s disease.

**Table 5:** Effect of exercise on subjects with sarcopenia and lung disease.

S.No	Authors, Year	Place, N	Subjects	Mean Age ± SD (Years), Sex	Exercise Intervention, Number of Groups	Measured Outcomes	Results	
1	Cebron Lipovec et al., 2016	Slovenia, 112	COPD patients (55% sarcopenia)	66.0 ± 8.0, Both M:F around 2:1	4-week short-term high-intensity pulmonary rehabilitation program, 2 groups: rehabilitation, control	-Blood biomarkers, SMI, IR	**sarcopenic compared to non sarcopenic group**	
							IR	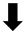
							fat mass index	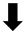
							waist circumference	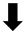
							LDL	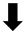

Note—N: number of subjects, COPD: Cardiac Obstructive Pulmonary Diseases, SMI: Skeletal Muscle Index, IR: Insulin Resistance, M: Male, LDL: Low density lipoprotein.

**Table 6:** Effect of exercise on subjects with sarcopenia and heart diseases.

**S.No**	**Authors, Year**	**Place, N**	**Subjects**	**Mean Age ± SD** **(Years), Sex**	**Exercise Intervention**	**Measured Outcomes**	**Results**	
1	Ogawa et al., 2021	Slovenia, 21	Cardiovascular surgery patients with sarcopenia defined by AWGS	69.6 ± 12.6, Both (M:F = 6:1)	low-intensity resistance training (RT) with moderate blood flow restriction (KAATSU RT), 2 Groups -control -KAATSU RT group * All patients had received a standard aerobic cardiac rehabilitation program. * The KAATSU RT group additionally executed low-intensity leg extension and leg press exercises with moderate blood flow restriction twice a week for 3 months. RT-intensity and volume were increased gradually	-MTH -SMI -handgrip strength -knee extensor strength -walking speed at baseline, 5–7 days after cardiac surgery, and after 3 months	**KAATSU RT compared to baseline.**	
							MTH	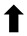
							SMI	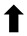
							walking speed	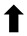
							knee extensor strength	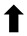

Note—MTH: anterior mid-thigh thickness, SMI: skeletal muscle mass index, CPK: Creatine phosphokinase, RT: resistance training, KAATSU RT: low-intensity resistance training with moderate blood flow restriction, M: Male, F: Female, * is the explanation of the interventions.

## Data Availability

Not applicable.
